# Life course socioeconomic position and DNA methylation age acceleration in mid-life

**DOI:** 10.1136/jech-2020-215608

**Published:** 2021-04-27

**Authors:** Anitha George, Rebecca Hardy, Juan Castillo Fernandez, Yvonne Kelly, Jane Maddock

**Affiliations:** 1 Department of Epidemiology & Public Health, UCL, London, UK; 2 UCL Social Research Institute, UCL, London, UK; 3 Department of Twin Research & Genetic Epidemiology, King's College London, London, UK; 4 MRC Unit for Lifelong Health and Ageing, Faculty of Population Health, UCL, London, UK

**Keywords:** ageing, health inequalities, social and life-course epidemiology, socio-economic

## Abstract

**Background:**

Ageing biomarkers can help us better understand how well-established socioeconomic position (SEP) disparities in ageing occur. A promising new set of DNAm methylation (DNAm)-based ageing biomarkers indicate through their age acceleration (AA) measures if biological ageing is slower or faster than chronological ageing. Few studies have investigated the association between SEP and DNAm AA.

**Methods:**

We used linear regression to examine the sex-adjusted relationships between childhood social class, adult social class, intergenerational social class change, education and adult household earnings with first (Horvath AA and Hannum AA) and second generation (PhenoAge AA and GrimAge AA) DNAm AA markers using data from the MRC National Survey of Health and Development.

**Results:**

In the first-generation biomarkers, there was little evidence of any associations with Horvath AA but associations of childhood social class and income with Hannum AA were observed. Strong associations were seen between greater disadvantage in childhood and adult SEP and greater AA in the second generation biomarkers. For example, those with fathers in an unskilled occupational social class in childhood had 3.6 years greater PhenoAge AA (95% CI 1.8 to 5.4) than those with fathers from a professional social class. Individuals without qualifications had higher AA compared with those with higher education (4.1 years greater GrimAge AA (95% CI 3.1 to 5.0)).

**Conclusion:**

Our findings highlight the importance of exposure to social disadvantage in childhood to the biological ageing process. The second generation clocks appear to be more sensitive to the accumulation of social disadvantage across the life course.

## Introduction

Socioeconomic differences in health have been reported across populations and age groups.^1 2^ A body of evidence links disadvantaged childhood and adult socioeconomic position (SEP) to adverse age-related outcomes, such as frailty.^3^ There is also evidence of a social gradient in health at older ages.^4^ Consequently, there is an ongoing interest in understanding how disadvantage translates to less favourable ageing processes.

Ageing is the time-related gradual deterioration of physiological function and greater susceptibility to death.^5^ Ageing biomarkers are defined as biological parameters that in the absence of disease, predict later age functional capability more effectively than chronological age.^6^ One promising set of ageing biomarkers are those calculated from DNA methylation (DNAm) levels at specific CpG (cytosine-phosphate-guanine) sites.^7^

The first generation of DNAm age biomarkers (the multitissue Horvath and blood-based Hannum clocks) were developed using penalised regression models which regressed chronological age on CpG sites, resulting in the selection of 353 and 71 CpG sites, respectively. However, they are only weakly associated with key clinical biomarkers^8^ leading to the development of two second generation blood-based DNAm biomarkers, PhenoAge and GrimAge, which incorporate information on individuals’ differing physiology and disease risk. PhenoAge, which includes 513 CpG sites, was estimated by regressing DNAm data on a phenotypic age predictor consisting of nine clinical biomarkers and chronological age.^9^ GrimAge incorporates DNAm-based surrogates of seven plasma proteins and smoking pack-years, as well as chronological age and sex, and comprises 1030 CpG sites.^10^ DNA methylation age acceleration (AA), that is, the deviation of DNAm age from chronological age, has been associated with mortality, greater risk of cardiovascular disease and cancer and worse physical function.^10^ The second generation markers, particularly GrimAge, appear to be stronger mortality predictors.^10^

The literature on the association between life course SEP and DNAm-based biomarkers is limited.^11^ To our knowledge, only five studies^12–16^ examined the association between childhood SEP and adult DNAm AA; they provide some evidence for an association of early life socioeconomic disadvantage and higher AA in the first generation markers.^13 14^ For education, which is the most investigated SEP indicator, lower attainment has been associated with increased AA in all four markers.^17–21^ For adult SEP indicators, no associations were found with adult social class and Horvath AA, Hannum AA or PhenoAge AA,^13 14 16^ nor between cross-sectional or cumulative employment status with Horvath AA, Hannum AA or PhenoAge AA.^12–14 16^ There was also mostly no evidence of an association between income and AA.^14 16 19 22^ The existing limited evidence on intergenerational social class change suggests an association with cumulative disadvantage and greater Horvath AA and Hannum AA^13 19^ but not PhenoAge AA.^16^

Most studies examined single SEP measures^15 17–24^ and of the five with early life measures, three used retrospectively collected indicators.^13 14 16^ Mainly studies considered relationships between SEP and the first generation biomarkers^12–15 17–20 22 24^ with few investigating PhenoAge AA and only one, to our knowledge, directly examining SEP associations with GrimAge AA.^16 21 23 25^

Our study, therefore, aimed to examine the association between life course SEP and Horvath AA, Hannum AA, PhenoAge AA and GrimAge AA biomarkers, measured at age 53 in a subsample of participants from the Medical Research Council (MRC) National Survey of Health and Development (NSHD). We examined the associations of occupational social class in childhood, adulthood and intergenerational change with each biomarker, and additionally considered education and adult household income. We hypothesised childhood to be a sensitive period for exposure to disadvantage in relation to DNAm AA, and that lower education would be most strongly associated with greater DNAm AA.

## Methods

### Study participants

The NSHD is a socially stratified cohort of 5362 singleton births in Britain in 1 week of March 1946. At age 53, data were collected from 3035 participants^26^ who compared with Census data were somewhat advantaged, but still broadly representative of UK-born individuals of the same age.^26^

Trained research nurses collected blood from 2759 of the age 53 participants^27^ from which DNAm was measured in 1376 individuals. This DNAm subsample was selected to minimise missing data on a range of health, social and age-related variables.

### DNAm data

The DNAm signals were measured using the Illumina Infinium MethylationEPIC Beadchip kit (Illumina, San Diego, California, USA). Standard quality control procedures were applied to the methylation data using ENmix in R and beta values were obtained using the noob normalisation method implemented in minfi in R. Signals with a detection p>1×10^−6^ and less than three beads were set to missing. Samples were excluded if they had missing data in more than 5% of the CpGs or if they were outliers, CpGs with missing data in more than 5% of the sample were excluded.

### DNA methylation age and white blood cells differential counts

The four DNAm-based biomarkers were calculated according to the methods outlined by Horvath (https://dnamage.genetics.ucla.edu/home) using the available software.^28^ We used DNAm AA which is the residual produced by regressing DNA age on chronological age, measured in units of years.^17^ White blood cell (WBC) differential count estimates of naïve and exhausted CD8+ T-lymphocytes, CD4+ T-lymphocytes, B cells, natural killer cells, monocytes and granulocytes were calculated simultaneously with the DNAm-based biomarkers.

### Socioeconomic position

We examined four indicators of SEP; childhood social class, indicated by father’s occupation when the study member was aged 4; own adult occupational social class at age 53; highest educational attainment to age 26 and household income at age 53.

Childhood and adult social class were categorised, according to the Registrar General’s six-level classification schema, as professional, intermediate, non-manual skilled, manual skilled, partly skilled and unskilled. A binary measure was derived which combined professional, intermediate and non-manual skilled into a non-manual category and the remaining groups into a manual category. Intergenerational social mobility was defined using the binary measures of social class and categorised as stable non-manual; non-manual to manual; manual to non-manual and stable manual across age four and 53. Educational attainment was categorised as higher education, school post-age 16, vocational education or school to age 16 and no qualifications. Annual net household income at age 53 was provided in bands and categorised as: £45 000 plus, £35 000–£44 999, £30 000–£34 999, £25 000–£29 000, £20 000–£24 999, £15 000–£19 999, £10 000–£14 999, less than £10 000.

### Statistical analysis

We fitted sex-adjusted regression models for childhood and adult social class separately with the four DNAm AA biomarkers. We tested for evidence of deviation from linearity across the six categories of social class. Where there was evidence of deviation from linearity, we tested for heterogeneity across groups, and where there was no evidence of a deviation, we fitted social class as a continuous variable and tested for a linear trend. As there is evidence that DNAm AA differs by sex^19^ and of SEP sex differences^29^ we tested for sex by social class interactions using the binary indicators.

We examined the association of the binary childhood and adult social class and intergenerational social mobility variables with each DNAm AA marker. We tested for a multiplicative relationship between childhood and adult social class and DNAm AA by testing for an interaction between the binary childhood and adult social class variables.

We investigated the association between education and, separately, household income and each DNAm AA marker using sex-adjusted models.

In response to a reviewer’s suggestion, we examined the association of childhood social class and the four DNAm AA biomarkers independent of education and adult social class by fitting models including all three variables.

We did not adjust for any other variables in our main analyses as we did not want to adjust for mediating factors, such as smoking, exercise and diet, which are known to be socially patterned and have been associated with DNAm AA.^20 30 31^ Similarly, WBC differential counts may be a mediator rather than a confounder between SEP and DNAm AA, thus their inclusion in the models may be an over-adjustment. However, WBC differential counts are correlated with DNAm AA^9^ and have often been adjusted for in comparable papers, therefore, we include models with this adjustment as sensitivity analyses. In additional, sensitivity analyses we applied weights to account for the social stratification in the original sample design.^26^ Finally, in response to a reviewer’s comments, we examined which of the GrimAge DNAm-based surrogate biomarkers had the strongest association with SEP. Here we used a z-score standardised transformation of the surrogate biomarkers to enable comparison between them and adjusted the models for sex. All analyses were conducted in Stata, V.15.

## Results

### Descriptive statistics

Table 1 shows the characteristics of the sample according to sex. Participants had a younger mean DNAm age than chronological age in all but GrimAge. The largest difference between DNAm age and chronological age was seen in PhenoAge where both sexes had DNAm age approximately 14 years lower than their chronological age. Women had a lower AA than men. The four AA measures had weak to moderate correlations among themselves (online supplemental table S1).

10.1136/jech-2020-215608.supp1 Supplementary data

**Table 1:** Descriptive statistics of sample with DNA methylation age acceleration measures at age 53 by sex (n=1376)

Variable	Men	Women
	Mean (SD)	Mean (SD)
Chronological age (year)	53.44 (0.16)	53.45 (0.18)
Horvath DNAm age (year)	50.69 (4.15)	49.61 (3.86)
Hannum DNAm age (year)	43.06 (4.28)	41.61 (3.95)
PhenoAge (year)	39.01 (5.59)	38.94 (5.61)
GrimAge (year)	57.99 (5.14)	55.32 (4.81)
Horvath age difference* (year)	−2.76 (4.15)	−3.84 (3.86)
Hannum age difference* (year)	−10.38 (4.28)	−11.84 (3.95)
PhenoAge age difference* (year)	−14.43 (5.60)	−14.51 (5.60)
GrimAge age difference* (year)	4.54 (5.14)	1.87 (4.80)
Horvath age acceleration† (year)	0.54 (4.15)	−0.54 (3.86)
Hannum age acceleration† (year)	0.79 (4.28)	−0.66 (3.95)
PhenoAge age acceleration† (year)	0.06 (5.59)	−0.02 (5.61)
GrimAge age acceleration† (year)	1.40 (5.14)	−1.28 (4.79)

	N (%)	N (%)
**Childhood social class (age 4**)		
Professional	36 (5.49)	41 (5.69)
Intermediate	115 (17.53)	121 (16.81)
Skilled non-manual	118 (17.99)	130 (18.06)
Skilled manual	203 (30.95)	221 (30.69)
Partly skilled	137 (20.88)	145 (20.14)
Unskilled	33 (5.03)	40 (5.56)
Missing	14 (2.13)	22 (3.06)
**Adults social class (age 53**)		
Professional	76 (11.59)	13 (1.81)
Intermediate	258 (39.33)	226 (31.39)
Skilled non-manual	73 (11.13)	232 (32.22)
Skilled manual	165 (25.15)	48 (6.67)
Partly skilled	53 (8.08)	103 (14.31)
Unskilled	15 (2.29)	43 (5.97)
Missing	16 (2.44)	55 (7.64)
**Intergenerational social class change**		
Stable non-manual	213 (32.47)	231 (32.08)
Non-manual to manual	49 (7.47)	51 (7.08)
Manual to non-manual	184 (28.05)	229 (31.81)
Stable manual	180 (27.44)	136 (18.89)
Missing	30 (4.57)	73 (10.14)
**Educational attainment (age 26**)		
Higher education	93 (14.18)	39 (5.42)
School post-16	193 (29.42)	156 (21.67)
Vocational/school to 16	146 (22.26)	251 (34.86)
No qualifications	223 (33.99)	260 (36.11)
Missing	1 (0.15)	14 (1.94)
**Annual household income (age 53**)		
£45 000 or more	73 (11.13)	51 (7.08)
£35 000–£44 999	63 (9.60)	54 (7.50)
£30 000–£34 999	103 (15.7)	78 (10.83)
£25 000–£29 999	58 (8.84)	59 (8.19)
£20 000–£24 999	99 (15.09)	111 (15.42)
£15 000–£19 999	99 (15.09)	95 (13.19)
£10 000–£14 999	82 (12.50)	121 (16.81)
Less than £10 000	61 (9.30)	108 (15.00)
Missing	18 (2.74)	43 (5.97)
**Total**	656	720

*Difference between DNAm age and chronological age.

†The residual from regressing DNA age on chronological age.

DNAm, DNAm methylation.

More women (32%) than men (28%) moved from a manual to non-manual class between age four and 53. Men had higher educational attainment than women with a greater proportion of them continuing education -after age 16 (44% of men compared with 27% of women).

### Childhood social class

Greater disadvantage in childhood social class was associated with higher Hannum AA, PhenoAge AA and GrimAge AA, but not Horvath AA (table 2). For Hannum AA and PhenoAge AA, there was evidence of a linear trend across the six social class groups. For GrimAge AA, compared with the professional group, greater AA was seen in the three manual categories. The greatest mean differences in AA were observed in PhenoAge AA where the unskilled social class group had 3.6 years (95% CI 1.8 to 5.4) higher PhenoAge AA than the professional group. The equivalent estimates for Hannum AA and GrimAge AA were 2 years (95% CI 0.7 to 3.4) and 3 years (95% CI 1.4 to 4.6) greater AA, respectively.

**Table 2:** Sex-adjusted regression models of the association of childhood social class (age 4) and adult social class (age 53), respectively, with four DNA methylation AA markers measured at age 53 in men and women (n=1273)

	Horvath AA	Hannum AA	PhenoAge AA	GrimAge AA
	Coeff. (95% CI)	Coeff. (95% CI)	Coeff. (95% CI)	Coeff. (95% CI)
**Childhood social class**				
Professional	Reference	Reference	Reference	Reference
Intermediate	1.02 (−0.05 to 2.08)	0.61 (−0.48 to 1.71)	1.69 (0.22 to 3.16)	1.26 (−0.02 to 2.54)
Skilled non-manual	0.42 (−0.63 to 1.47)	0.81 (−0.27 to 1.89)	1.83 (0.38 to 3.29)	0.85 (−0.42 to 2.12)
Skilled manual	0.60 (−0.41 to 1.61)	1.27 (0.24 to 2.30)	2.69 (1.30 to 4.07)	2.55 (1.34 to 3.76)
Partly skilled	0.45 (−0.59 to 1.50)	1.34 (0.26 to 2.41)	2.23 (0.78 to 3.67)	2.14 (0.88 to 3.40)
Unskilled	1.00 (−0.33 to 2.33)	2.04 (0.68 to 3.41)	3.59 (1.75 to 5.43)	2.95 (1.35 to 4.56)
P value for trend	0.94	<0.001	<0.001	<0.001*
**Adult social class**				
Professional	Reference	Reference	Reference	Reference
Intermediate	−0.20 (−1.15 to 0.74)	0.43 (−0.54 to 1.40)	0.41 (−0.91 to 1.72)	0.89 (−0.24 to 2.03)
Skilled non-manual	0.06 (−0.96 to 1.07)	0.91 (−0.14 to 1.95)	1.13 (−0.28 to 2.54)	1.45 (0.23 to 2.67)
Skilled manual	0.84 (−0.18 to 1.86)	1.28 (0.23 to 2.33)	1.18 (−0.24 to 2.60)	3.01 (1.78 to 4.24)
Partly skilled	−0.33 (−1.42 to 0.77)	−0.11 (−1.23 to 1.02)	1.12 (−0.40 to 2.64)	2.16 (0.84 to 3.47)
Unskilled	−0.52 (−1.91 to 0.87)	0.58 (−0.85 to 2.00)	2.25 (0.32 to 4.18)	3.70 (2.04 to 5.37)
P value for trend	0.04*	0.01*	0.01	<0.001*

*Test for heterogeneity across groups if evidence of deviation from linearity.

AA, age acceleration.

### Adult social class

Disadvantaged adult social class was associated with greater PhenoAge AA and GrimAge AA (table 2). For GrimAge AA, the manual classes had higher mean AA than the non-manual categories. For Hannum AA and Horvath AA there was some variation in AA across categories but no clear trend.

### Binary social class and intergenerational social class change

Results were similar for the binary indicators of social class as for the six category variables (online supplemental table S2). For childhood social class, the manual group had higher mean AA than the non-manual group for Hannum AA (0.7 years, 95% CI 0.3 to 1.2), PhenoAge AA (1.1 years, 95% CI 0.5 to 1.7) and GrimAge AA (1.5 years, 95% CI 1.0 to 2.1), but not Horvath AA. For adult social class, there were differences for PhenoAge AA (0.7 years AA, 95% CI 0.1 to 1.4) and GrimAge AA (1.8 years AA, 95% CI 1.2 to 2.4) but no evidence of association for Hannum AA or Horvath AA. There was no evidence of a consistent interaction between the binary social class indicators and sex.

For intergenerational social class change and DNAm AA (table 3), there was no evidence of an interaction between childhood and adult social class, suggesting an additive association. For PhenoAge AA and GrimAge AA, mobile individuals had a mean AA between the stable non-manual and stable manual group. Those from a more disadvantaged childhood social class had higher Hannum AA and PhenoAge AA, regardless of their adult social class. There was no evidence of a relationship with Horvath AA.

**Table 3:** Sex-adjusted regression models of the association of intergenerational social class change (between age 4 and age 53) with four DNA methylation AA markers measured at age 53 in men and women (n=1273)

	Horvath AA	Hannum AA	PhenoAge AA	GrimAge AA
	Coeff. (95% CI)	Coeff. (95% CI)	Coeff. (95% CI)	Coeff. (95% CI)
**Intergenerational social class change**				
Stable non-manual	Reference	Reference	Reference	Reference
Non-manual to manual	0.76 (−0.11 to 1.64)	0.45 (−0.45 to 1.34)	0.63 (−0.58 to 1.84)	1.74 (0.69 to 2.78)
Manual to non-manual	0.06 (−0.48 to 0.60)	0.98 (0.43 to 1.53)	1.05 (0.30 to 1.79)	1.27 (0.62 to 1.92)
Stable manual	0.19 (−0.40 to 0.77)	0.62 (0.03 to 1.22)	1.39 (0.58 to 2.19)	2.62 (1.92 to 3.32)

AA, age acceleration.

### Educational attainment and household income

Lower educational attainment was associated with greater PhenoAge AA and GrimAge AA, but not Horvath AA or Hannum AA (table 4). For GrimAge AA, the greatest mean difference was between individuals without qualifications compared with those with higher education (4.1 years AA (95% CI 3.1 to 5.0). Lower income was associated with increased AA in all but Horvath AA (table 4). Associations were linear with Hannum AA and PhenoAge AA, while for GrimAge AA greater mean AA is seen in the three lowest income groups compared with the highest earning group. There was no evidence of sex by education or by income interactions.

**Table 4:** Sex-adjusted regression models of the association of highest educational attainment (age 26) and household income (age 53) separately, with four DNA methylation AA markers measured at age 53 in men and women (n=1361 and 1315, respectively)

	Horvath AA	Hannum AA	PhenoAge AA	GrimAge AA
	Coeff. (95% CI)	Coeff. (95% CI)	Coeff. (95% CI)	Coeff. (95% CI)
**Educational attainment**				
Higher education	Reference	Reference	Reference	Reference
School post16	−0.40 (−1.21 to 0.40)	0.24 (−0.59 to 1.07)	0.35 (−0.77 to 1.47)	1.45 (0.49 to 2.42)
Vocational/school to 16	−0.39 (−1.19 to 0.41)	0.70 (−0.12 to 1.53)	1.16 (0.05 to 2.28)	2.44 (1.47 to 3.40)
No qualifications	−0.28 (−1.06 to 0.49)	0.36 (−0.44 to 1.16)	1.21 (0.12 to 2.29)	4.06 (3.13 to 5.00)
P value for trend	0.80	0.38	0.01	<0.001
**Household income**				
£45 000 or more	Reference	Reference	Reference	Reference
£35 000–£44 999	0.64 (−0.38 to 1.66)	0.74 (−0.30 to 1.77)	1.06 (−0.35 to 2.46)	1.32 (0.10 to 2.54)
£30 000–£34 999	0.28 (−0.65 to 1.20)	0.74 (−0.19 to 1.68)	0.74 (−0.52 to 2.01)	0.64 (−0.46 to 1.75)
£25 000–£29 999	0.53 (−0.49 to 1.55)	0.27 (−0.77 to 1.31)	−0.02 (−1.42 to 1.39)	0.16 (−1.06 to 1.38)
£20 000–£24 999	0.65 (−0.25 to 1.54)	0.87 (−0.04 to 1.79)	1.39 (0.16 to 2.62)	1.38 (0.30 to 2.45)
£15 000–£19 999	0.35 (−0.56 to 1.26)	0.96 (0.03 to 1.89)	1.79 (0.54 to 3.04)	2.59 (1.51 to 3.68)
£10 000–£14 999	0.17 (−0.73 to 1.08)	0.73 (−0.19 to 1.65)	1.48 (0.24 to 2.73)	2.53 (1.45 to 3.61)
Less than £10 000	0.69 (−0.25 to 1.63)	1.46 (0.50 to 2.41)	2.37 (1.07 to 3.66)	3.73 (2.60 to 4.86)
P value for trend	0.56	0.01	<0.001	<0.001*

*Test for heterogeneity across groups if evidence of deviation from linearity.

AA, age acceleration.

### Childhood social class adjusted for adult SEP

Childhood social class disadvantage remained associated with greater Hannum AA and PhenoAge AA after adjusting for adult social class and both adult social class and educational attainment. The association with GrimAge remained after adjusting for adult social class but was substantially attenuated after additionally adjusting for educational attainment (online supplemental table S3).

### Sensitivity analyses

Adjustments for WBC differential counts resulted in a large degree of attenuation in all models (online supplemental tables S4–S8). However, the associations remained between childhood social class and PhenoAge AA and GrimAge AA, and between adult social class, education and income with GrimAge AA. Applying the stratification weights did not change the conclusions (online supplemental tables S9–S14).

Associations were seen between the four SEP indicators and the DNAm surrogates of smoking pack-years, adrenomedullin, beta-2-microglobulin (B2M), cystatin-C and tissue inhibitor of metalloproteinases 1 (TIMP-1). Associations were stronger for pack-years, notably so for educational attainment. Relatively strong associations were seen between the four SEP indicators and TIMP-1, as well as between adult social class and B2M, education and growth differentiation factor 15 (GDF-15) and income and B2M and cystatin-C. No associations were found between any measure of SEP and DNAm leptin (online supplemental tables S15–S18).

## Discussion

We found that childhood SEP was associated with Hannum AA, PhenoAge AA and GrimAge AA in mid-life, but not with Horvath AA. The association with adult social class was weaker than for childhood social class for Hannum AA and PhenoAge AA but similar in GrimAge AA. For intergenerational social mobility, individuals from a more advantaged childhood social class had lower Hannum AA and PhenoAge AA regardless of their adult social class while there was evidence of accumulation for GrimAge AA. Education was associated with PhenoAge AA and GrimAge AA but not Horvath AA or Hannum AA. Income showed similar relationships to adult social class. The importance of childhood SEP for Hannum AA and PhenoAge AA was further supported as associations remained after adjusting for adult social class and education. There was no evidence of social variation in Horvath AA.

Similar to our findings, a previous UK study (n=1094) found earlier life disadvantage to be associated with greater Hannum AA.^14^ However, they also found an association with Horvath AA.^14^ Unlike our findings, no association was found with PhenoAge AA in a group of Irish residents aged 50 plus^16^ however, the study had less statistical power due to the smaller sample (n=490). Both these previous studies used retrospective measures of father’s social class, which is subject to recall bias.

Examining intergenerational mobility provided some support for the hypothesis that childhood is a sensitive period for exposure to disadvantaged social class for Hannum AA and PhenoAge AA. For GrimAge AA, and to a lesser extent PhenoAge AA, there was a suggestion of an accumulative effect of disadvantage. Existing studies found cumulative social class disadvantage to be associated with greater Hannum AA but not PhenoAge AA.^16 19^ Our results for PhenoAge and Grim Age are in line with the literature on gradient constraint which posits that individuals’ health is shaped by their social class of origin as well as their class of destination.^32^ Thereby those moving from a less to a more advantaged social class have better health than the class they left but worse than the one they enter.^32^

Our finding that lower educational attainment was associated with greater PhenoAge AA and GrimAge AA is akin to two studies which similarly showed dose response relationships with these same biomarkers.^21 23^ Four papers, to our knowledge, have examined the association between income and Hannum AA, Horvath AA and PhenoAge AA and only one found an association with Hannum AA in the same direction as our results.^14 16 19 22^ We found that education was particularly strongly associated with GrimAge in models also including childhood and adult social class suggesting that it may be more important than accumulation of social class. However, educational attainment measured in early adulthood is strongly influenced by parental characteristics, including early life social class, making it difficult to fully disentangle the separate influences of social class accumulation and educational attainment.

The estimated effect sizes between the most and least disadvantaged found in our study are not trivial and have important health implications. For example, a 1-year increase in GrimAge AA was associated with a 10% increased mortality hazard.^33^

The results provide some indication of early life programming of DNAm AA. Exposure to adversity in early childhood has been shown to lead to persistent DNAm alterations.^34^ A possible mechanism is stress as those who experienced early life stress have exhibited differential methylation levels associated with long-term dysregulation of the hypothalamic–pituitary–adrenal axis, indicating potential childhood origins of adult disease.^35^ Extant research, mainly examining first generation AA biomarkers, signals that early life rather than adult SEP is more important for adult DNAm AA. However, our study indicates that the second generation clocks are more sensitive to SEP disparities across the life course and thus, childhood exposures and possible mediators, such as adult diet and other socially patterned exposures associated with DNAm AA^25 36^ could explain observed associations of adult SEP with PhenoAge and GrimAge.

As the AA biomarkers were estimated using machine learning methods, their biological significance is not clear. However, the Hannum clock and PhenoAge are associated with genes related to immune function and pro-inflammatory signalling pathways.^37 38^ While our finding of no association of SEP with Horvath AA aligns with the evidence that it is a marker of intrinsic cell ageing mostly unrelated to lifestyle factors.^38^

Potential biological pathways through which SEP is associated with GrimAge AA may be seen in the consistent association of the four SEP indicators with DNAm surrogates of smoking pack-years and TIMP-1. The association in the former is less surprising considering the strong social patterning of smoking behaviour^39^ and the identified relationship between smoking and methylation levels.^40^ In contrast, TIMP-1 has only recently been found to be key in the pathology of several human diseases.^41^ Interestingly, its DNAm surrogate was found to have stronger associations with several adverse physical and cognitive outcomes than the other GrimAge DNAm surrogates in middle-aged and older individuals.^42^ However, SEP associations were found with other GrimAge DNAm surrogates and the importance of the individual surrogates in comparison to the whole biomarker is unclear.

The substantial degree of attenuation seen in all models after adjusting for WBC differential counts suggests they may be a mediator rather than confounder. There is evidence of SEP differences in WBC counts, with advantage associated with better immune cell profiles^43^ and changes in differential counts are markers of age-associated conditions, such as immunoscenesence.^8^ In our models, attenuation was greatest for PhenoAge AA. The Hannum clock and PhenoAge reflect age-associated changes in the composition of cells and tissue, and WBC counts are used in the estimation of the latter, thus, the adjustment could remove some of their biological meaning.^37^

We saw a noticeable underestimation of chronological age in the Hannum clock and PhenoAge. Our sample is not unusually healthy (online supplemental tables S19 and S20) and other studies show the two DNAm age biomarkers to underestimate chronological age, with comparably large differences seen by Zhao *et al*.^14 21 23 44^ Importantly, due to their method of estimation our measures of AA should not produce biased results.^15^ The AA measures are not guaranteed to be correlated^38^ and we, like others,^18 20 21^ found low to moderate correlations between the markers.

Our results may also indicate the importance of tissue specificity for DNAm AA as no association was found between SEP and DNAm AA measured in buccal cells of 790 NSHD women of the same age.^12 15^

### Strengths and limitations

Our study, to the best of our knowledge, is the first to investigate the association of life course SEP using the four DNAm AA markers and is the first to investigate SEP GrimAge AA associations in UK data. The SEP measures were collected prospectively minimising recall bias. As we used data from a birth cohort, we do not have to disentangle the cohort and period effects related to the SEP indicators.

The limitations include the inability to generalise to the current UK population as the original sample is representative of the British-born population before major migration flows to the UK. However, the age 53 sample are broadly representative of the UK-born population of a similar age.^26^ There was no evidence of differences by sex, childhood and adult social class, education and several health-related indicators between those with and without DNAm AA data (online supplemental tables S19 and S20). For income, the DNAm AA sample were somewhat over-represented in the middle income categories. The DNAm samples were chosen to minimise missing data on key variables, such as SEP. Therefore, if a third variable is associated with SEP and DNAm AA and influenced participation, this could result in collider bias.^45^ Finally, adjustment for WBC differential counts appears to be important, but we only have estimated counts.

## Conclusion

Our study suggests the importance of exposure to social disadvantage in childhood in the ageing process as well as a need to understand the role of socially patterned behaviours across the life course, which as potential mediators in the relationship between SEP and DNAm AA may add to accelerated biological ageing. The second generation clocks appear to be more sensitive to disadvantage after early life with some indication that the accumulation of social class disadvantage is more strongly associated with PhenoAge and educational attainment with GrimAge AA, but further investigation is required to replicate our findings.

**What is already known on this subject**

- Existing studies showed that socioeconomic disadvantage in childhood and lower educational attainment, but not adult socioeconomic position, are associated with greater biological ageing measured by DNA methylation-based ageing biomarkers.
- This research mainly examined the association between single measures of socioeconomic position and first-generation DNA methylation-based ageing biomarkers.

**What this study adds**

- The study looked at life course socioeconomic position in both first-generation and second-generation DNA methylation-based biomarkers. Results from the latter indicate that disadvantage across the life course, not only childhood and early adulthood, is associated with greater biological ageing.

## Data Availability

Data may be obtained from a third party and are not publicly available. Data used in this publication are available to bona fide researchers upon request to the NSHD Data Sharing Committee via a standard application procedure. Further details can be found at http://www.nshd.mrc.ac.uk/data doi: 10.5522/NSHD/S201.
