# Supplementary material for: Life course socioeconomic position and DNA methylation age acceleration in mid-life
Source: J Epidemiol Community Health. 2021 Apr 27;75(11):1084–90. doi: 10.1136/jech-2020-215608 (PMC8515099; doi:10.1136/jech-2020-215608)
Supplement: Supplementary data [file jech-2020-215608supp001.pdf]

Table S1

Pairwise correlation between the four DNAm age acceleration markers (n=1376)

	**Horvath AA**	**Hannum AA**	**PhenoAge AA**	**GrimAge AA**
**Horvath AA**	1.00			
**Hannum AA**	0.40	1.00		
**PhenoAge AA**	0.42	0.49	1.00	
**GrimAge AA**	0.13	0.25	0.41	1.00

AA Age acceleration

Table S2

Adjusted regression models of the association of childhood social class (age 4) and adult social class (age 53), respectively, with four DNA methylation age acceleration markers measured at age 53 in men and women (n=1273)

	**Horvath AA[a]**	**Hannum AA[a]**	**PhenoAge AA[a]**	**GrimAge AA[a]**	**Hannum AA[b]**	**Hannum AA[b]**	**PhenoAge AA[b]**	**GrimAge AA[b]**
**Childhood Social class**	Coeff. (95% CI)	Coeff. (95% CI)	Coeff. (95% CI)	Coeff. (95% CI)	Coeff. (95% CI)	Coeff. (95% CI)	Coeff. (95% CI)	Coeff. (95% CI)
Non-manual	Reference	Reference	Reference	Reference	Reference	Reference	Reference	Reference
Manual	-0.03 (-0.48, 0.42)	0.74 (0.28, 1.20)	1.08 (0.46, 1.70)	1.53 (0.99, 2.07)	-0.13 (-0.57, 0.32)	0.38 (-0.02, 0.78)	0.48 (-0.05, 1.01)	1.10 (0.59, 1.61)
LRT p-value[c]	0.05	0.43	0.28	0.17				
	**Horvath AA[a]**	**Hannum AA[a]**	**PhenoAge AA[a]**	**GrimAge AA[a]**	**Horvath AA[b]**	**Hannum AA[b]**	**PhenoAge AA[b]**	**GrimAge AA[b]**
**Adult social class**	Coeff. (95% CI)	Coeff. (95% CI)	Coeff. (95% CI)	Coeff. (95% CI)	Coeff. (95% CI)	Coeff. (95% CI)	Coeff. (95% CI)	Coeff. (95% CI)
Non-manual	Reference	Reference	Reference	Reference	Reference	Reference	Reference	Reference
Manual	0.30 (-0.17, 0.77)	0.11 (-0.38, 0.60)	0.70 (0.05, 1.36)	1.80 (1.23, 2.37)	0.30 (-0.17, 0.76)	-0.04 (-0.46, 0.39)	0.40 (-0.15, 0.96)	1.51 (0.98, 2.04)
LRT p-value[d]	0.96	0.56	0.56	0.46				

AA age acceleration, LRT log-likelihood ratio test
[a] Adjusted for sex
[b] Adjusted for sex, naïve and exhausted CD8+T-lymphocytes, CD4+ T-lymphocytes, B cells, natural killer cells, monocytes and granulocytes
[c] P-value log-likelihood ratio test for interaction between binary childhood social class (age 4) and sex
[d] P-value log-likelihood ratio test for interaction between binary adult class (age 53) and sex

Table S3

Adjusted regression models of the association of childhood social class (age 4) with four DNA methylation age acceleration markers measured at age 53 in men and women (n=1261)

	Horvath AA[a]	Horvath AA[b]	Hannum AA[a]	Hannum AA[b]	PhenoAge AA[a]	PhenoAge AA[b]	GrimAge AA[a]	GrimAge AA[b]
**Father's Social Class**	Coeff. (95% CI)	Coeff. (95% CI)	Coeff. (95% CI)	Coeff. (95% CI)	Coeff. (95% CI)	Coeff. (95% CI)	Coeff. (95% CI)	Coeff. (95% CI)
Professional	Reference	Reference	Reference	Reference	Reference	Reference	Reference	Reference
Intermediate	0.97 (-0.10, 2.03)	1.05 (-0.02, 2.12)	0.57 (-0.52, 1.67)	0.62 (-0.48, 1.72)	1.59 (0.12, 3.05)	1.57 (0.09, 3.05)	1.07 (-0.20, 2.35)	0.69 (-0.57, 1.96)
Skilled non-manual	0.43 (-0.62, 1.49)	0.54 (-0.52, 1.61)	0.82 (-0.27, 1.91)	0.87 (-0.22, 1.97)	1.68 (0.22, 3.14)	1.68 (0.20, 3.15)	0.68 (-0.59, 1.94)	0.26 (-1.00, 1.53)
Skilled manual	0.45 (-0.57, 1.47)	0.62 (-0.43, 1.68)	1.20 (0.15, 2.25)	1.37 (0.28, 2.45)	2.41 (1.00, 3.81)	2.43 (0.98, 3.89)	2.00 (0.78, 3.23)	1.09 (-0.15, 2.34)
Partly Skilled	0.25 (-0.83, 1.32)	0.42 (-0.69, 1.53)	1.27 (0.17, 2.37)	1.47 (0.33, 2.60)	1.96 (0.48, 3.44)	2.00 (0.48, 3.53)	1.38 (0.09, 2.66)	0.41 (-0.89, 1.72)
Unskilled	0.65 (-0.71, 2.00)	0.82 (-0.57, 2.20)	1.84 (0.44, 3.23)	2.09 (0.66, 3.51)	3.02 (1.15, 4.89)	3.09 (1.18, 5.01)	2.07 (0.45, 3.69)	0.97 (-0.67, 2.61)
P-value for trend	0.56	0.83	0.00	<0.001	0.00	0.00	<0.001[c]	0.44

AA age acceleration
[a] Adjusted for sex and adult social class
[b] Adjusted for sex, adult social class and education
[c] Test for heterogeneity across groups if evidence of deviation from linearity

Table S4

Adjusted regression models of the association between childhood social class (age 4) with four DNA methylation age acceleration markers measured at age 53 in men and women (n=1273)

	**Horvath AA[a]**	**Hannum AA[a]**	**PhenoAge AA[a]**	**GrimAge AA[a]**
**Childhood social class**	Coeff. (95% CI)	Coeff. (95% CI)	Coeff. (95% CI)	Coeff. (95% CI)
Professional	Reference	Reference	Reference	Reference
Intermediate	0.93 (-0.12, 1.98)	0.42 (-0.53, 1.38)	1.17 (-0.09, 2.42)	1.00 (-0.20, 2.21)
Skilled non-manual	0.24 (-0.80, 1.28)	0.30 (-0.64, 1.25)	0.75 (-0.50, 2.00)	0.27 (-0.92, 1.47)
Skilled manual	0.41 (-0.59, 1.41)	0.63 (-0.28, 1.53)	1.42 (0.22, 2.61)	1.76 (0.62, 2.91)
Partly Skilled	0.25 (-0.78, 1.29)	0.76 (-0.18, 1.70)	1.08 (-0.16, 2.32)	1.42 (0.23, 2.60)
Unskilled	0.55 (-0.78, 1.87)	0.83 (-0.37, 2.03)	1.54 (-0.05, 3.12)	1.78 (0.26, 3.30)
P-value for trend	0.53	0.07	0.10	<0.001[b]

AA age acceleration

[a] Adjusted for sex, naïve and exhausted CD8+T-lymphocytes, CD4+ T-lymphocytes, B cells, natural killer cells, monocytes and granulocytes

[b] Test for heterogeneity across groups if evidence of deviation from linearity

Table S5

Adjusted regression models of the association between own adult social class (age 53) with four DNA methylation age acceleration markers measured at age 53 in men and women (n=1273)

	**Horvath AA[a]**	**Hannum AA[a]**	**PhenoAge AA[a]**	**GrimAge AA[a]**
**Adult social class**	Coeff. (95% CI)	Coeff. (95% CI)	Coeff. (95% CI)	Coeff. (95% CI)
Professional	Reference	Reference	Reference	Reference
Intermediate	-0.36 (-1.29, 0.58)	-0.03 (-0.88, 0.81)	-0.37 (-1.49, 0.76)	0.58 (-0.48, 1.65)
Skilled non-manual	-0.10 (-1.11, 0.91)	0.42 (-0.49, 1.33)	0.22 (-0.98, 1.43)	1.13 (-0.02, 2.28)
Skilled manual	0.71 (-0.30, 1.72)	0.66 (-0.25, 1.58)	0.08 (-1.13, 1.29)	2.30 (1.15, 3.46)
Partly Skilled	-0.39 (-1.47, 0.69)	-0.40 (-1.37, 0.58)	0.51 (-0.79, 1.81)	1.82 (0.59, 3.06)
Unskilled	-0.93 (-2.31, 0.44)	-0.58 (-1.82, 0.67)	0.29 (-1.36, 1.95)	3.00 (1.43, 4.57)
P-value for trend	0.02[b]	0.02[b]	0.08	<0.001

AA age acceleration
[a] Adjusted for sex, naïve and exhausted CD8+T-lymphocytes, CD4+ T-lymphocytes, B cells, natural killer cells, monocytes and granulocytes
[b] Test for heterogeneity across groups if evidence of deviation from linearity

Table S6

Adjusted regression models of the association of intergenerational social class change (between age 4 and age 53) with four DNA methylation age acceleration markers measured at age 53 in men and women (n=1273)

	**Horvath AA[a]**	**Hannum AA[a]**	**PhenoAge AA[a]**	**GrimAge AA[a]**
**Intergenerational social class change**	Coeff. (95% CI)	Coeff. (95% CI)	Coeff. (95% CI)	Coeff. (95% CI)
Stable non-manual	Reference	Reference	Reference	Reference
Non-manual to manual	0.69 (-0.17, 1.55)	0.42 (-0.36, 1.20)	0.47 (-0.57, 1.50)	1.63 (0.65, 2.61)
Manual to non-manual	-0.08 (-0.62, 0.45)	0.64 (0.15, 1.12)	0.48 (-0.16, 1.12)	0.91 (0.30, 1.52)
Stable manual	0.11 (-0.46, 0.69)	0.22 (-0.30, 0.74)	0.69 (-0.00, 1.38)	2.06 (1.40, 2.71)

AA age acceleration

[a] Adjusted for sex, naïve and exhausted CD8+T-lymphocytes, CD4+ T-lymphocytes, B cells, natural killer cells, monocytes and granulocytes

Table S7

Adjusted regression models of the association of highest educational attainment (age 26) with four DNA methylation age acceleration markers measured at age 53 in men and women (n=1361)

	**Horvath AA[a]**	**Hannum AA[a]**	**PhenoAge AA[a]**	**GrimAge AA[a]**
**Educational attainment**	Coeff. (95% CI)	Coeff. (95% CI)	Coeff. (95% CI)	Coeff. (95% CI)
Higher education	Reference	Reference	Reference	Reference
School post 16	-0.44 (-1.24, 0.35)	0.11 (-0.60, 0.83)	0.08 (-0.89, 1.04)	1.24 (0.34, 2.14)
Vocational/school to 16	-0.49 (-1.28, 0.31)	0.37 (-0.35, 1.08)	0.57 (-0.39, 1.53)	2.03 (1.13, 2.93)
No quals	-0.41 (-1.19, 0.36)	-0.20 (-0.90, 0.50)	0.26 (-0.67, 1.20)	3.40 (2.53, 4.28)
P-value for trend	0.52	0.31	0.46	<0.001

AA Age acceleration
[a] Adjusted for sex, naïve and exhausted CD8+T-lymphocytes, CD4+ T-lymphocytes, B cells, natural killer cells, monocytes and granulocytes

Table S8

Adjusted regression models of the association of annual household income (age 53) with four DNA methylation age markers measured at age 53 in men and women (n=1315)

	**Horvath AA**[a]	**Hannum AA**[a]	**PhenoAge AA**[a]	**GrimAge AA**[a]
**Household income**	Coeff. (95% CI)	Coeff. (95% CI)	Coeff. (95% CI)	Coeff. (95% CI)
£45 000 or more	Reference	Reference	Reference	Reference
£35 000 - £44 999	0.61 (-0.39, 1.62)	0.34 (-0.57, 1.25)	0.06 (-1.16, 1.27)	0.71 (-0.43, 1.86)
£30 000 - £34 999	0.13 (-0.78, 1.04)	0.23 (-0.59, 1.04)	0.01 (-1.09, 1.10)	0.26 (-0.77, 1.29)
£25 000 - £29 999	0.58 (-0.42, 1.59)	0.19 (-0.71, 1.10)	-0.22 (-1.43, 0.99)	0.02 (-1.13, 1.16)
£20 000 - £24 999	0.53 (-0.36, 1.42)	0.23 (-0.57, 1.03)	0.18 (-0.89, 1.25)	0.59 (-0.41, 1.60)
£15 000 - £19 999	0.22 (-0.68, 1.12)	0.26 (-0.55, 1.07)	0.57 (-0.51, 1.66)	1.86 (0.84, 2.88)
£10 000 - £14 999	0.12 (-0.78, 1.02)	0.08 (-0.73, 0.89)	0.25 (-0.83, 1.33)	1.66 (0.64, 2.68)
Less than £10 000	0.48 (-0.45, 1.42)	0.56 (-0.28, 1.40)	0.83 (-0.30, 1.96)	2.90 (1.84, 3.96)
P-value for trend	0.84	0.52	0.09	<0.001[b]

AA age acceleration
[a] Adjusted for sex, naïve and exhausted CD8+T-lymphocytes, CD4+ T-lymphocytes, B cells, natural killer cells, monocytes and granulocytes
[b] Test for heterogeneity across groups if evidence of deviation from linearity

Table S9

Weighted[a] regression models of the association between childhood social class (age 4) with four DNA methylation age acceleration markers measured at age 53 in men and women (n=1273)

	**Horvath AA[b]**	**Hannum AA[b]**	**PhenoAge AA[b]**	**GrimAge AA[b]**
**Childhood social class**	Coeff. (95% CI)	Coeff. (95% CI)	Coeff. (95% CI)	Coeff. (95% CI)
Professional	Reference	Reference	Reference	Reference
Intermediate	1.03 (-0.04, 2.09)	0.62 (-0.48, 1.72)	1.77 (0.30, 3.25)	1.23 (-0.05, 2.52)
Skilled non-manual	0.42 (-0.63, 1.48)	0.82 (-0.27, 1.90)	1.85 (0.39, 3.30)	0.85 (-0.42, 2.12)
Skilled manual	0.63 (-0.46, 1.73)	1.29 (0.17, 2.41)	3.05 (1.54, 4.56)	2.44 (1.12, 3.76)
Partly Skilled	0.47 (-0.60, 1.54)	1.35 (0.25, 2.44)	2.40 (0.93, 3.87)	2.09 (0.80, 3.37)
Unskilled	1.03 (-0.37, 2.43)	2.06 (0.63, 3.49)	3.94 (2.02, 5.87)	2.85 (1.16, 4.53)
	**Horvath AA[c]**	**Hannum AA[c]**	**PhenoAge AA[c]**	**GrimAge AA[c]**
**Childhood social class**	Coeff. (95% CI)	Coeff. (95% CI)	Coeff. (95% CI)	Coeff. (95% CI)
Professional	Reference	Reference	Reference	Reference
Intermediate	0.95 (-0.11, 2.00)	0.44 (-0.52, 1.40)	1.26 (-0.01, 2.52)	0.99 (-0.22, 2.20)
Skilled non-manual	0.24 (-0.80, 1.28)	0.31 (-0.64, 1.25)	0.77 (-0.48, 2.01)	0.27 (-0.92, 1.47)
Skilled manual	0.47 (-0.61, 1.56)	0.70 (-0.28, 1.68)	1.82 (0.53, 3.12)	1.72 (0.47, 2.96)
Partly Skilled	0.28 (-0.77, 1.34)	0.79 (-0.16, 1.75)	1.27 (0.01, 2.53)	1.40 (0.19, 2.61)
Unskilled	0.61 (-0.78, 1.99)	0.90 (-0.36, 2.15)	1.93 (0.27, 3.59)	1.73 (0.14, 3.32)

AA age acceleration
[a] Weighted using the NSHD social stratification weights to take account of the sample design
[b] Adjusted for sex
[c] Adjusted for sex, naïve and exhausted CD8+T-lymphocytes, CD4+ T-lymphocytes, B cells, natural killer cells, monocytes and granulocytes

Table S10

Weighted[a] regression models of the association between adult social class (age 53) with four DNA methylation age acceleration markers measured at age 53 in men and women (n=1273)

	**Horvath AA[b]**	**Hannum AA[b]**	**PhenoAge AA[b]**	**GrimAge AA[b]**
**Adult social class**	Coeff. (95% CI)	Coeff. (95% CI)	Coeff. (95% CI)	Coeff. (95% CI)
Professional	Reference	Reference	Reference	Reference
Intermediate	-0.20 (-1.15, 0.74)	0.43 (-0.54, 1.40)	0.41 (-0.91, 1.72)	0.90 (-0.23, 2.04)
Skilled non-manual	0.06 (-0.96, 1.08)	0.88 (-0.16, 1.92)	1.10 (-0.31, 2.51)	1.38 (0.16, 2.60)
Skilled manual	0.85 (-0.18, 1.87)	1.22 (0.17, 2.27)	1.13 (-0.30, 2.55)	2.86 (1.63, 4.09)
Partly Skilled	-0.33 (-1.42, 0.77)	-0.14 (-1.26, 0.99)	1.09 (-0.43, 2.61)	2.08 (0.77, 3.40)
Unskilled	-0.51 (-1.90, 0.88)	0.52 (-0.91, 1.95)	2.19 (0.26, 4.12)	3.55 (1.88, 5.22)
	**Horvath AA[c]**	**Hannum AA[c]**	**PhenoAge AA[c]**	**GrimAge AA[c]**
**Adult social class**	Coeff. (95% CI)	Coeff. (95% CI)	Coeff. (95% CI)	Coeff. (95% CI)
Professional	Reference	Reference	Reference	Reference
Intermediate	-0.36 (-1.29, 0.57)	-0.03 (-0.88, 0.81)	-0.37 (-1.49, 0.76)	0.59 (-0.48, 1.65)
Skilled non-manual	-0.09 (-1.10, 0.92)	0.41 (-0.50, 1.33)	0.23 (-0.98, 1.44)	1.08 (-0.07, 2.22)
Skilled manual	0.73 (-0.28, 1.75)	0.65 (-0.27, 1.56)	0.09 (-1.13, 1.31)	2.20 (1.05, 3.36)
Partly Skilled	-0.38 (-1.46, 0.70)	-0.40 (-1.38, 0.58)	0.51 (-0.79, 1.81)	1.77 (0.54, 3.01)
Unskilled	-0.91 (-2.29, 0.46)	-0.59 (-1.84, 0.66)	0.30 (-1.36, 1.96)	2.90 (1.33, 4.47)

AA age acceleration

[a] Weighted using the NSHD social stratification weights to take account of the sample design

[b] Adjusted for sex

[c] Adjusted for sex, naïve and exhausted CD8+T-lymphocytes, CD4+ T-lymphocytes, B cells, natural killer cells, monocytes and granulocytes

Table S11

Weighted[a] regression models of the association between intergenerational social class change (age 53) with four DNA methylation age acceleration markers measured at age 53 in men and women (n=1273)

	**Horvath AA[b]**	**Hannum AA[b]**	**PhenoAge AA[b]**	**GrimAge AA[b]**
**Intergenerational social class change**	Coeff. (95% CI)	Coeff. (95% CI)	Coeff. (95% CI)	Coeff. (95% CI)
Stable non-manual	Reference	Reference	Reference	Reference
Non-manual to manual	0.76 (-0.11, 1.64)	0.45 (-0.45, 1.34)	0.63 (-0.58, 1.84)	1.73 (0.69, 2.78)
Manual to non-manual	0.01 (-0.60, 0.62)	0.99 (0.36, 1.62)	1.16 (0.31, 2.01)	1.08 (0.34, 1.81)
Stable manual	0.14 (-0.50, 0.79)	0.64 (-0.03, 1.30)	1.49 (0.60, 2.38)	2.44 (1.67, 3.21)
	**Horvath AA[c]**	**Hannum AA[c]**	**PhenoAge AA[c]**	**GrimAge AA[c]**
**Intergenerational social class change**	Coeff. (95% CI)	Coeff. (95% CI)	Coeff. (95% CI)	Coeff. (95% CI)
Stable non-manual	Reference	Reference	Reference	Reference
Non-manual to manual	0.69 (-0.17, 1.55)	0.42 (-0.36, 1.20)	0.47 (-0.56, 1.50)	1.63 (0.65, 2.61)
Manual to non-manual	-0.11 (-0.72, 0.49)	0.70 (0.15, 1.24)	0.64 (-0.09, 1.37)	0.77 (0.09, 1.46)
Stable manual	0.09 (-0.55, 0.72)	0.28 (-0.30, 0.85)	0.84 (0.08, 1.61)	1.93 (1.21, 2.65)

AA age acceleration
[a] Weighted using the NSHD social stratification weights to take account of the sample design
[b] Adjusted for sex
[c] Adjusted for sex, naïve and exhausted CD8+T-lymphocytes, CD4+ T-lymphocytes, B cells, natural killer cells, monocytes and granulocytes

Table S12

Weighted[a] regression models of the association of childhood social class (age 4) and adult social class (age 53), respectively, with four DNA methylation age acceleration markers measured at age 53 in men and women (n=1273)

	**Horvath AA[b]**	**Hannum AA[b]**	**PhenoAge AA[b]**	**GrimAge AA[b]**	**Horvath AA[c]**	**Hannum AA[c]**	**PhenoAge AA[c]**	**GrimAge AA[c]**
**Childhood Social Class**	Coeff. (95% CI)	Coeff. (95% CI)	Coeff. (95% CI)	Coeff. (95% CI)	Coeff. (95% CI)	Coeff. (95% CI)	Coeff. (95% CI)	Coeff. (95% CI)
Non-manual	Reference	Reference	Reference	Reference	Reference	Reference	Reference	Reference
Manual	-0.07 (-0.60, 0.46)	0.75 (0.21, 1.29)	1.19 (0.46, 1.92)	1.36 (0.72, 2.00)	-0.15 (-0.68, 0.37)	0.43 (-0.04, 0.90)	0.64 (0.02, 1.27)	0.98 (0.38, 1.58)
	**Horvath AA[b]**	**Hannum AA[b]**	**PhenoAge AA[b]**	**GrimAge AA[b]**	**Horvath AA[c]**	**Hannum AA[c]**	**PhenoAge AA[c]**	**GrimAge AA[c]**
**Adult Social Class**	Coeff. (95% CI)	Coeff. (95% CI)	Coeff. (95% CI)	Coeff. (95% CI)	Coeff. (95% CI)	Coeff. (95% CI)	Coeff. (95% CI)	Coeff. (95% CI)
Non-manual	Reference	Reference	Reference	Reference	Reference	Reference	Reference	Reference
Manual	0.30 (-0.18, 0.77)	0.06 (-0.43, 0.55)	0.66 (-0.01, 1.32)	1.68 (1.11, 2.26)	0.30 (-0.17, 0.78)	-0.06 (-0.48, 0.37)	0.41 (-0.16, 0.97)	1.44 (0.90, 1.97)

AA age acceleration

[a] Weighted using the NSHD social stratification weights to take account of the sample design

[b] Adjusted for sex

[c] Adjusted for sex, naïve and exhausted CD8+T-lymphocytes, CD4+ T-lymphocytes, B cells, natural killer cells, monocytes and granulocytes

Table S13

Weighted[a] regression models of the association between educational attainment (age 53) with four DNA methylation age acceleration markers measured at age 53 in men and women (n=1361)

	**Horvath AA[b]**	**Hannum AA[b]**	**PhenoAge AA[b]**	**GrimAge AA[b]**
**Educational attainment**	Coeff. (95% CI)	Coeff. (95% CI)	Coeff. (95% CI)	Coeff. (95% CI)
Higher education	Reference	Reference	Reference	Reference
School post 16	-0.41 (-1.22, 0.40)	0.20 (-0.64, 1.03)	0.31 (-0.82, 1.43)	1.40 (0.43, 2.36)
Vocational/school to 16	-0.40 (-1.22, 0.41)	0.62 (-0.22, 1.45)	1.09 (-0.04, 2.21)	2.32 (1.35, 3.30)
No qualifications	-0.30 (-1.10, 0.49)	0.23 (-0.59, 1.05)	1.09 (-0.02, 2.20)	3.90 (2.95, 4.86)
	**Horvath AA[c]**	**Hannum AA[c]**	**PhenoAge AA[c]**	**GrimAge AA[c]**
**Educational attainment**	Coeff. (95% CI)	Coeff. (95% CI)	Coeff. (95% CI)	Coeff. (95% CI)
Higher education	Reference	Reference	Reference	Reference
School post 16	-0.44 (-1.24, 0.35)	0.10 (-0.62, 0.81)	0.08 (-0.89, 1.04)	1.22 (0.31, 2.12)
Vocational/school to 16	-0.48 (-1.28, 0.32)	0.34 (-0.39, 1.06)	0.57 (-0.40, 1.54)	1.98 (1.07, 2.89)
No qualifications	-0.41 (-1.20, 0.38)	-0.25 (-0.96, 0.47)	0.27 (-0.69, 1.23)	3.33 (2.43, 4.23)

AA age acceleration
[a] Weighted using the NSHD social stratification weights to take account of the sample design
[b] Adjusted for sex
[c] Adjusted for sex, naïve and exhausted CD8+T-lymphocytes, CD4+ T-lymphocytes, B cells, natural killer cells, monocytes and granulocytes

Table S14

Weighted[a] regression models of the association between household income in pounds/year (age 53) with four DNA methylation age acceleration markers measured at age 53 in men and women (n=1315)

	**Horvath AA[b]**	**Hannum AA[b]**	**PhenoAge AA[b]**	**GrimAge AA[b]**
**Household income**	Coeff. (95% CI)	Coeff. (95% CI)	Coeff. (95% CI)	Coeff. (95% CI)
£45,000 or more	Reference	Reference	Reference	Reference
£35,000 - £44,999	0.64 (-0.38, 1.66)	0.73 (-0.31, 1.77)	1.05 (-0.35, 2.45)	1.30 (0.09, 2.52)
£30,000 - £34,999	0.28 (-0.65, 1.20)	0.73 (-0.20, 1.67)	0.73 (-0.54, 2.00)	0.62 (-0.49, 1.72)
£25,000 - £29,999	0.53 (-0.49, 1.56)	0.27 (-0.77, 1.31)	-0.02 (-1.42, 1.38)	0.15 (-1.06, 1.37)
£20,000 - £24,999	0.64 (-0.26, 1.54)	0.84 (-0.07, 1.76)	1.35 (0.12, 2.59)	1.29 (0.22, 2.37)
£15,000 - £19,999	0.35 (-0.56, 1.26)	0.93 (0.00, 1.86)	1.75 (0.50, 3.01)	2.51 (1.42, 3.60)
£10,000 - £14,999	0.17 (-0.74, 1.07)	0.70 (-0.22, 1.62)	1.45 (0.20, 2.70)	2.45 (1.37, 3.53)
Less than £10,000	0.69 (-0.26, 1.63)	1.42 (0.46, 2.37)	2.32 (1.02, 3.62)	3.62 (2.49, 4.75)
	**Horvath AA[c]**	**Hannum AA[c]**	**PhenoAge AA[c]**	**GrimAge AA[c]**
**Household income**	Coeff. (95% CI)	Coeff. (95% CI)	Coeff. (95% CI)	Coeff. (95% CI)
£45,000 or more	Reference	Reference	Reference	Reference
£35,000 - £44,999	0.62 (-0.39, 1.62)	0.34 (-0.57, 1.25)	0.06 (-1.16, 1.27)	0.71 (-0.44, 1.85)
£30,000 - £34,999	0.13 (-0.78, 1.04)	0.23 (-0.59, 1.04)	0.01 (-1.09, 1.10)	0.25 (-0.78, 1.28)
£25,000 - £29,999	0.58 (-0.42, 1.59)	0.19 (-0.71, 1.10)	-0.22 (-1.43, 0.99)	0.01 (-1.12, 1.15)
£20,000 - £24,999	0.53 (-0.36, 1.42)	0.23 (-0.57, 1.03)	0.18 (-0.89, 1.25)	0.55 (-0.46, 1.55)
£15,000 - £19,999	0.22 (-0.68, 1.13)	0.26 (-0.55, 1.07)	0.57 (-0.51, 1.66)	1.81 (0.79, 2.83)
£10,000 - £14,999	0.13 (-0.77, 1.03)	0.08 (-0.73, 0.89)	0.25 (-0.83, 1.34)	1.62 (0.60, 2.64)
Less than £10,000	0.49 (-0.45, 1.43)	0.56 (-0.28, 1.40)	0.83 (-0.30, 1.96)	2.84 (1.78, 3.90)

AA age acceleration
[a] Weighted using the NSHD social stratification weights to take account of the sample design
[b] Adjusted for sex
[c] Adjusted for sex, naïve and exhausted CD8+T-lymphocytes, CD4+ T-lymphocytes, B cells, natural killer cells, monocytes and granulocytes

Table 15

Sex-adjusted regression models of the association of childhood social class (age 4) with the eight z-score standardised GrimAge DNA methylation-based surrogate biomarkers measured at age 53 in men and women (n=1273)

	DNAm packyrs[a]	DNAm ADM[a]	DNAm B2M[a]	DNAm Cystatin C[a]	DNAm GDF-15[a]	DNAm Leptin[a]	DNAm PAI-1[a]	DNAm TIMP-1[a]
**Childhood social class**	Coeff. (95% CI)	Coeff. (95% CI)	Coeff. (95% CI)	Coeff. (95% CI)	Coeff. (95% CI)	Coeff. (95% CI)	Coeff. (95% CI)	Coeff. (95% CI)
Professional	Reference	Reference	Reference	Reference	Reference	Reference	Reference	Reference
Intermediate	0.17 (-0.09, 0.42)	0.12 (-0.07, 0.32)	0.18 (-0.09, 0.44)	0.15 (-0.12, 0.41)	0.27 (0.01, 0.54)	0.05 (-0.10, 0.19)	0.07 (-0.17, 0.30)	0.32 (0.07, 0.57)
Skilled non-manual	0.16 (-0.10, 0.41)	0.08 (-0.12, 0.27)	-0.02 (-0.27, 0.24)	0.08 (-0.18, 0.34)	0.14 (-0.12, 0.41)	0.06 (-0.08, 0.21)	0.00 (-0.23, 0.24)	0.10 (-0.15, 0.35)
Skilled manual	0.43 (0.18, 0.67)	0.17 (-0.02, 0.35)	0.23 (-0.02, 0.48)	0.23 (-0.02, 0.48)	0.42 (0.17, 0.67)	0.07 (-0.06, 0.21)	0.19 (-0.03, 0.42)	0.41 (0.17, 0.65)
Partly Skilled	0.35 (0.09, 0.60)	0.12 (-0.07, 0.31)	0.21 (-0.05, 0.47)	0.23 (-0.03, 0.49)	0.28 (0.02, 0.54)	0.02 (-0.13, 0.16)	0.16 (-0.07, 0.39)	0.41 (0.16, 0.66)
Unskilled	0.48 (0.16, 0.81)	0.32 (0.07, 0.56)	0.26 (-0.07, 0.58)	0.41 (0.08, 0.74)	0.37 (0.04, 0.70)	-0.05 (-0.23, 0.13)	0.17 (-0.12, 0.47)	0.32 (0.00, 0.64)
P-value for trend	<0.001	0.04	0.02[b]	0.01	0.00[b]	0.64	0.03	<0.001[b]

DNAmpackyrs DNA methylation-based surrogate of smoking pack-years
DNAm ADM DNA methylation-based surrogate of adrenomedullin levels
DNAm B2M DNA methylation-based surrogate of beta-2 microglobulin
DNAm Cystatin C DNA methylation-based surrogate of cystatin C
DNAm GDF-15 DNA methylation-based surrogate of growth differentiation factor 15
DNAm Leptin DNA methylation-based surrogate of leptin
DNAm PAI-1 DNA methylation-based surrogate of plasminogen activation inhibitor 1
DNAm TIMP-1 DNA methylation-based surrogate of tissue inhibitor metalloproteinase 1
[a] Standardised variable
[b] Test for heterogeneity across groups if evidence of deviation from linearity

Table 16

Sex-adjusted regression models of the association of adult social class (age 53) with the eight z-score standardised GrimAge DNA methylation-based surrogate biomarkers measured at age 53 in men and women (n=1273)

	DNAm packyrs[a]	DNAm ADM[a]	DNAm B2M[a]	DNAm Cystatin C[a]	DNAm GDF-15[a]	DNAm Leptin[a]	DNAm PAI-1[a]	DNAm TIMP-1[a]
**Adult social class**	Coeff. (95% CI)	Coeff. (95% CI)	Coeff. (95% CI)	Coeff. (95% CI)	Coeff. (95% CI)	Coeff. (95% CI)	Coeff. (95% CI)	Coeff. (95% CI)
Professional	Reference	Reference	Reference	Reference	Reference	Reference	Reference	Reference
Intermediate	0.11 (-0.12, 0.34)	0.14 (-0.04, 0.31)	0.23 (-0.01, 0.46)	0.08 (-0.16, 0.31)	0.16 (-0.08, 0.40)	0.11 (-0.02, 0.24)	0.03 (-0.18, 0.24)	0.18 (-0.05, 0.41)
Skilled non-manual	0.18 (-0.07, 0.42)	0.27 (0.09, 0.46)	0.35 (0.10, 0.60)	0.19 (-0.06, 0.44)	0.07 (-0.18, 0.33)	0.11 (-0.03, 0.25)	0.01 (-0.22, 0.23)	0.29 (0.05, 0.54)
Skilled manual	0.51 (0.26, 0.76)	0.21 (0.02, 0.40)	0.48 (0.23, 0.73)	0.35 (0.10, 0.60)	0.34 (0.08, 0.59)	0.09 (-0.05, 0.23)	0.11 (-0.12, 0.34)	0.41 (0.17, 0.66)
Partly Skilled	0.40 (0.13, 0.66)	0.22 (0.01, 0.42)	0.24 (-0.03, 0.51)	0.06 (-0.21, 0.33)	0.16 (-0.11, 0.44)	0.15 (-0.00, 0.30)	-0.05 (-0.30, 0.19)	0.31 (0.05, 0.57)
Unskilled	0.65 (0.31, 0.98)	0.32 (0.06, 0.57)	0.62 (0.28, 0.96)	0.48 (0.14, 0.82)	0.20 (-0.15, 0.54)	0.17 (-0.02, 0.36)	0.07 (-0.24, 0.38)	0.44 (0.10, 0.77)
P-value for trend	<0.001	0.01	<0.001[b]	<0.001[b]	0.11	0.16	0.93	<0.001

DNAmpackyrs DNA methylation-based surrogate of smoking pack-years
DNAm ADM DNA methylation-based surrogate of adrenomedullin levels
DNAm B2M DNA methylation-based surrogate of beta-2 microglobulin
DNAm Cystatin C DNA methylation-based surrogate of cystatin C
DNAm GDF-15 DNA methylation-based surrogate of growth differentiation factor 15
DNAm Leptin DNA methylation-based surrogate of leptin
DNAm PAI-1 DNA methylation-based surrogate of plasminogen activation inhibitor 1
DNAm TIMP-1 DNA methylation-based surrogate of tissue inhibitor metalloproteinase 1
[a] Standardised variable
[b] Test for heterogeneity across groups if evidence of deviation from linearity

Table 17

Sex-adjusted regression models of the association of highest educational attainment (age 26) with the eight z-score standardised GrimAge DNA methylation-based surrogate biomarkers measured at age 53 in men and women (n=1361)

	DNAm packyrs[a]	DNAm ADM[a]	DNAm B2M[a]	DNAm Cystatin C[a]	DNAm GDF-15[a]	DNAm Leptin[a]	DNAm PAI-1[a]	DNAm TIMP-1[a]
**Educational attainment**	Coeff. (95% CI)	Coeff. (95% CI)	Coeff. (95% CI)	Coeff. (95% CI)	Coeff. (95% CI)	Coeff. (95% CI)	Coeff. (95% CI)	Coeff. (95% CI)
Higher education	Reference	Reference	Reference	Reference	Reference	Reference	Reference	Reference
School post 16	0.33 (0.14, 0.53)	0.00 (-0.15, 0.15)	0.10 (-0.10, 0.30)	0.10 (-0.10, 0.30)	0.09 (-0.11, 0.29)	-0.00 (-0.11, 0.11)	0.00 (-0.18, 0.19)	0.07 (-0.12, 0.27)
Vocational/school to 16	0.51 (0.32, 0.70)	0.03 (-0.12, 0.18)	0.24 (0.04, 0.44)	0.14 (-0.06, 0.34)	0.20 (-0.00, 0.39)	0.01 (-0.10, 0.12)	0.12 (-0.06, 0.30)	0.20 (0.00, 0.39)
No qualifications	0.83 (0.65, 1.02)	0.12 (-0.03, 0.26)	0.28 (0.08, 0.47)	0.29 (0.10, 0.49)	0.39 (0.20, 0.58)	0.01 (-0.09, 0.12)	0.13 (-0.04, 0.31)	0.35 (0.16, 0.54)
P-value for trend	<0.001	0.02	0.00	<0.001	<0.001	0.68	0.03	<0.001

DNAmpackyrs DNA methylation-based surrogate of smoking pack-years
DNAm ADM DNA methylation-based surrogate of adrenomedullin levels
DNAm B2M DNA methylation-based surrogate of beta-2 microglobulin
DNAm Cystatin C DNA methylation-based surrogate of cystatin C
DNAm GDF-15 DNA methylation-based surrogate of growth differentiation factor 15
DNAm Leptin DNA methylation-based surrogate of leptin
DNAm PAI-1 DNA methylation-based surrogate of plasminogen activation inhibitor 1
DNAm TIMP-1 DNA methylation-based surrogate of tissue inhibitor metalloproteinase 1
[a] Standardised variable

Table 18

Sex-adjusted regression models of the association of household income (age 53) with the eight z-score standardised GrimAge DNA methylation-based surrogate biomarkers measured at age 53 in men and women (n=1315)

	DNAm packyrs[a]	DNAm ADM[a]	DNAm B2M[a]	DNAm Cystatin C[a]	DNAm GDF-15[a]	DNAm Leptin[a]	DNAm PAI-1[a]	DNAm TIMP-1[a]
**Household income**	Coeff. (95% CI)	Coeff. (95% CI)	Coeff. (95% CI)	Coeff. (95% CI)	Coeff. (95% CI)	Coeff. (95% CI)	Coeff. (95% CI)	Coeff. (95% CI)
£45,000 or more	Reference	Reference	Reference	Reference	Reference	Reference	Reference	Reference
£35,000 - £44,999	0.24 (0.00, 0.49)	0.11 (-0.08, 0.30)	0.10 (-0.15, 0.35)	0.12 (-0.13, 0.38)	0.17 (-0.08, 0.43)	0.03 (-0.11, 0.17)	-0.03 (-0.26, 0.20)	0.16 (-0.09, 0.40)
£30,000 - £34,999	0.09 (-0.13, 0.31)	0.00 (-0.17, 0.17)	0.23 (0.01, 0.46)	0.07 (-0.16, 0.29)	0.04 (-0.19, 0.27)	-0.04 (-0.17, 0.08)	0.12 (-0.09, 0.33)	0.10 (-0.12, 0.32)
£25,000 - £29,999	0.01 (-0.23, 0.25)	-0.03 (-0.22, 0.16)	0.03 (-0.22, 0.28)	0.09 (-0.17, 0.34)	0.03 (-0.23, 0.28)	0.00 (-0.14, 0.14)	0.02 (-0.21, 0.25)	0.10 (-0.14, 0.35)
£20,000 - £24,999	0.23 (0.02, 0.45)	0.07 (-0.09, 0.24)	0.35 (0.13, 0.57)	0.16 (-0.06, 0.38)	0.09 (-0.13, 0.31)	0.06 (-0.06, 0.19)	0.04 (-0.17, 0.24)	0.18 (-0.04, 0.39)
£15,000 - £19,999	0.46 (0.25, 0.68)	0.19 (0.02, 0.35)	0.37 (0.14, 0.59)	0.29 (0.07, 0.52)	0.26 (0.04, 0.49)	0.10 (-0.02, 0.22)	0.01 (-0.20, 0.21)	0.36 (0.15, 0.58)
£10,000 - £14,999	0.47 (0.25, 0.68)	0.15 (-0.02, 0.31)	0.29 (0.07, 0.52)	0.28 (0.06, 0.50)	0.22 (-0.00, 0.45)	-0.00 (-0.13, 0.12)	0.09 (-0.12, 0.30)	0.27 (0.05, 0.49)
Less than £10,000	0.67 (0.45, 0.89)	0.25 (0.08, 0.43)	0.46 (0.23, 0.69)	0.41 (0.17, 0.64)	0.37 (0.14, 0.61)	0.09 (-0.04, 0.22)	0.19 (-0.03, 0.40)	0.39 (0.16, 0.62)
P-value for trend	0.00[b]	0.00	<0.001	<0.001	0.00	0.12	0.12	<0.001

DNAmpackyrs DNA methylation-based surrogate of smoking pack-years
DNAm ADM DNA methylation-based surrogate of adrenomedullin levels
DNAm B2M DNA methylation-based surrogate of beta-2 microglobulin
DNAm Cystatin C DNA methylation-based surrogate of cystatin C
DNAm GDF-15 DNA methylation-based surrogate of growth differentiation factor 15
DNAm Leptin DNA methylation-based surrogate of leptin

DNAm PAI-1 DNA methylation-based surrogate of plasminogen activation inhibitor 1
DNAm TIMP-1 DNA methylation-based surrogate of tissue inhibitor metalloproteinase 1
[a] Standardised variable
[b] Test for heterogeneity across groups if evidence of deviation from linearity

Table S19

Descriptive statistics of the age 53 sample by DNAm AA data availability

**DNAm AA data**	No	Yes	All
Variable	N (%)	N (%)	N (%)
**Sex**			
Men	816 (49.19)	656 (47.67)	1472 (48.5)
Women	843 (50.81)	720 (52.33)	1563 (51.5)
Total	1659 (100)	1376 (100)	3035 (100)
P-value chi-squared test			0.41
**Childhood social class (age 4)**			
Professional	87 (6.03)	77 (5.75)	164 (5.89)
Intermediate	251 (17.39)	236 (17.61)	487 (17.5)
Skilled non-manual	288 (19.96)	248 (18.51)	536 (19.26)
Skilled manual	422 (29.24)	424 (31.64)	846 (30.4)
Partly Skilled	292 (20.24)	282 (21.04)	574 (20.63)
Unskilled	103 (7.14)	73 (5.45)	176 (6.32)
Total	1443 (100)	1340 (100)	2783 (100)
P-value chi-squared test			0.35
**Adult social class (age 53)**			
Professional	118 (8.02)	89 (6.82)	207 (7.46)
Intermediate	560 (38.07)	484 (37.09)	1044 (37.61)
Skilled non-manual	316 (21.48)	305 (23.37)	621 (22.37)
Skilled manual	279 (18.97)	213 (16.32)	492 (17.72)
Partly Skilled	147 (9.99)	156 (11.95)	303 (10.91)
Unskilled	51 (3.47)	58 (4.44)	109 (3.93)
Total	1471 (100)	1305 (100)	2776 (100)
P-value chi-squared test			0.09
**Educational attainment (age 26)**			
Higher education	148 (9.83)	132 (9.7)	280 (9.77)
School post 16	381 (25.32)	349 (25.64)	730 (25.47)
Vocational/school to	404 (26.84)	397 (29.17)	801 (27.95)
No qualifications	572 (38.01)	483 (35.49)	1055 (36.81)
Total	1505 (100)	1361 (100)	2866 (100)
P-value chi-squared test			0.45
**Household income (£/yr) (age 53)**			
45,000 or more	189 (12.47)	124 (9.43)	313 (11.06)
35,000 - 44,999	158 (10.42)	117 (8.9)	275 (9.71)
30,000 - 34,999	197 (12.99)	181 (13.76)	378 (13.35)
25,000 - 29,999	110 (7.26)	117 (8.9)	227 (8.02)
20,000 - 24,999	204 (13.46)	210 (15.97)	414 (14.62)
15,000 - 19,999	233 (15.37)	194 (14.75)	427 (15.08)
10,000 - 14,999	209 (13.79)	203 (15.44)	412 (14.55)
Less than 10,000	216 (14.25)	169 (12.85)	385 (13.6)
Total	1516 (100)	1315 (100)	2831 (100)
P-value chi-squared test			0.03
**Smoking history upto age 53**			
Current smoker	362 (22.44)	333 (24.22)	695 (23.26)
Ex-smoker	788 (48.85)	630 (45.82)	1418 (47.46)
Never smoker	463 (28.7)	412 (29.96)	875 (29.28)
Total	1613 (100)	1375 (100)	2988 (100)
P-value chi-squared test			0.24

**Physical activity in last 4 weeks (age 53)**			
None	807 (50.06)	670 (48.76)	1477 (49.46)
1-4 times	265 (16.44)	253 (18.41)	518 (17.35)
5 or more times	540 (33.5)	451 (32.82)	991 (33.19)
Total	1612 (100)	1374 (100)	2986 (100)
P-value chi-squared test			0.36
	Mean (N)	Mean (N)	Mean (N)
**Body mass index (age 53)**	27.49 (1578)	27.35 (1370)	27.43 (2948)
P-value t-test			0.41
**Forced expiratory volume - 1 second[a] (age 53)**	2.8 (1533)	2.76 (1326)	2.78 (2859)
P-value t-test			0.17
**Grip strength (age 53)**	37.55 (1527)	37.59 (1323)	37.57 (2850)
P-value t-test			0.94
**Diastolic blood pressure (age 53)**	84.32 (1573)	84.57 (1354)	84.44 (2927)
P-value t-test			0.58
**Systolic blood pressure (age 53)**	136 (1573)	136.17 (1354)	136.08 (2927)
P-value t-test			0.41

AA age acceleration

[a] Forced expiratory volume is an indicator of lung function

Table S20

Logistic regression of the association of SEP and health indicators and DNAm AA data availability[a] in the age 53 sample

Variable	OR (95% CI)	N
**Sex**		
Male	Reference	
Female	1.06 (0.92, 1.23)	3035
**Childhood social class (age 4)**		
Professional	Reference	
Intermediate	1.06 (0.75, 1.51)	
Skilled non-manual	0.97 (0.69, 1.38)	
Skilled manual	1.14 (0.81, 1.59)	
Partly Skilled	1.09 (0.77, 1.54)	
Unskilled	0.80 (0.52, 1.23)	2783
**Adult social class (age 53)**		
Professional	Reference	
Intermediate	1.15 (0.85, 1.55)	
Skilled non-manual	1.28 (0.93, 1.76)	
Skilled manual	1.01 (0.73, 1.40)	
Partly Skilled	1.41 (0.99, 2.01)	
Unskilled	1.51 (0.95, 2.40)	2776
**Education attainment (age 26)**		
Higher education	Reference	
School post16	1.03 (0.78, 1.35)	
Vocational/school to 16	1.10 (0.84, 1.45)	
No quals	0.95 (0.73, 1.23)	2866
**Household income £/year (age 53)**		
£45 000 or more	Reference	
£35,000 - £44,999	1.13 (0.81, 1.57)	
£30,000 - £34,999	1.40 (1.03, 1.90)	
£25,000 - £29,999	1.62 (1.15, 2.29)	
£20,000 - £24,999	1.57 (1.17, 2.11)	
£15,000 - £19,999	1.27 (0.94, 1.71)	
£10,000 - £14,999	1.48 (1.10, 1.99)	
Less than £10,000	1.19 (0.88, 1.61)	2831
**Smoking history up to age 53**		
Current smoker	Reference	
Ex-smoker	0.87 (0.72, 1.04)	
Never	0.97 (0.79, 1.18)	2988
**Physical activity in last 4 weeks (age 53)**		
None	Reference	
1-4 times a week	1.15 (0.94, 1.41)	
5 or more times	1.01 (0.86, 1.18)	2986
**Body mass index in kg/(m2) (age 53)**		
Mean	0.99 (0.98, 1.01)	2948
**Forced expiratory volume in 1 second[b] (age 53)**		
Mean	0.93 (0.84, 1.03)	2859
**Grip strength (age 53)**		
Mean	1.00 (1.00, 1.01)	2850
**Diastolic blood pressure (age 53)**		

Mean	1.00 (1.00, 1.01)	2927
**Systolic blood pressure (age 53)**		
Mean	1.00 (1.00, 1.00)	2927

[a] DNAm data availability: 0 DNAm data not collected, 1 DNAm data available

[b] Forced expiratory volume is an indicator of lung function
